# Influence of Rigid Polyurethane Foam Production Technology on Cryogenic Water Uptake

**DOI:** 10.3390/polym17121669

**Published:** 2025-06-16

**Authors:** Vladimir Yakushin, Vanesa Dhalivala, Laima Vevere, Ugis Cabulis

**Affiliations:** Latvian State Institute of Wood Chemistry, Dzerbenes Street 27, LV-1006 Riga, Latvia; vladimir.yakushin@kki.lv (V.Y.); vanesa.dhalivala@kki.lv (V.D.); laima.vevere@kki.lv (L.V.)

**Keywords:** polyurethane foam, cryogenic insulation, moisture uptake, thermal conductivity, liquid nitrogen

## Abstract

This study explores how production technology influences spray-applied rigid polyurethane (PUR) foam insulation’s cryogenic performance. In cryogenic applications such as liquid gas storage, insulation must minimise heat transfer and resist moisture ingress under severe thermal gradients. Experimental aluminium vessels were insulated with PUR foam of varying thicknesses and surface conditions—rough, machined smooth, and with a urea-based protective coating—and then tested using dynamic boil-off of liquid nitrogen (LN_2_). Foam properties, including adhesion, mechanical strength, thermal expansion, thermal conductivity, and closed-cell content, were evaluated. The results revealed that thicker insulation reduced both effective thermal conductivity and moisture uptake. Although the urea-coated vessel showed minimal water absorption, the coating increased overall thermal conductivity due to its heat conduction and condensation behaviour. Moisture was primarily absorbed near the foam surface, and no cumulative effects were observed during repeated tests. The effective thermal conductivity was determined by interpolating boil-off data, confirming that insulation performance strongly depends on thickness, surface condition, and environmental humidity. These findings provide valuable guidance for the design and application of PUR foam insulation in cryogenic environments.

## 1. Introduction

Evaluation of the effectiveness of cryogenic insulation, which is frequently used to insulate tanks of liquid oxygen (LO_2_) and liquid hydrogen (LH_2_) as well as to store and transport liquefied natural gas. Among the various types of cryogenic insulation considered in the works [1,2,3,4] and others, and considering factors such as heat leaks, protection from water vapour penetration, avoidance of condensation and frost and ice formation, and weight and cost factors, polyurethane and polyisocyanurate foams occupy an important place.

Cryogenic foam insulation must have sufficient mechanical strength and good adhesion to the metal substrate (for sprayed foam). A lower thermal coefficient of linear expansion should help reduce shrinkages and cracking of the insulating material during cooling. Additionally, foam insulation should be dimensionally stable, low-flammable for locations that must adhere to strict fire safety regulations, and highly resistant to corrosion and UV rays, such as in offshore applications [5].

However, the determining requirement for cryogenic insulation is the heat leakage rate through the insulation. The thermal conductivity value determines these losses. The thermal conductivity value depends on various factors., i.e., temperature, density of foam and moisture content, and the type of gas contained in foam cells. Therefore, moisture permeability and the associated cryogenic moisture uptake under actual use conditions are essential parameters for cryogenic insulation [6].

Both thermal conductivity and other properties of polymer foam are not constant and change during ageing and weathering of cryogenic insulation. Therefore, the final stage of cryogenic insulation assessment is environmental exposure testing.

Despite the straightforward theoretical basis of thermal conductivity measurements, their practical execution under cryogenic conditions introduces substantial technical and non-technical challenges, leading to various established experimental configurations for determining effective thermal conductivity. A review of experimental methods on insulation thermal conductivity measurements at cryogenic temperatures is presented in [7]. Among the numerous steady-state measurements is the Guarded Hot Plate approach. It is based on measuring the electrical power that provides the required temperature gradient between the plates [8]. The primary idea behind the Boil-off Calorimetry method is to measure the rate at which the cryogenic liquid evaporates or boils off in the tank’s inner chamber in order to calculate heat flow Q [3,9,10,11]. The tank’s outside is maintained at a higher temperature. A gas flow meter or weight scale is used to measure the boil-off flow rate, which is directly proportional to the energy that the heat of vaporisation transfers through the test specimen.

There was previously little knowledge on how moisture can seep into the foam under significant temperature gradients when one side of the foam is exposed to the temperatures of liquid gases and the other side is exposed to room temperatures and high relative humidity. In order to study this phenomenon, specialised equipment was created. The impact of several variables on the amount of cryogenic moisture absorption was examined in [6,12,13,14].

This effect becomes most critical when the liquid gas in the tank is hydrogen, and the cryogenic insulation has not only a certain number of open cells, but also various defects in the form of voids or channels. Then this phenomenon is called cryopumping. When a void in a substance or structure is sufficiently cold to densify a contained volume of air gases, this phenomenon is known as cryopumping [12]. A vacuum is produced as the gases condense; adjacent gases are drawn or pumped into the void if there is a conduit to the surface or adjacent voids. The densified gases expand and are pumped or ejected from the void when the material or structure is heated. The material may sustain the most damage during this cryopumping expansion phase. If the gases are densified into a void, the void is heated quickly, but the flow of gases from the void is constrained, leading to a rapid build-up of pressure; cryopumping can then be problematic for cryogenic materials and structures. The potential for cryopumping may be indicated by high cryogenic water uptake levels.

The EU has defined the goal of increasing energy independence [15], which logically includes a directive on the promotion of the use of energy from renewable sources [16] and the hydrogen strategy [17]. Special emphasis in EU planning documents is placed on Green hydrogen—which is characterised by the fact that it is produced by electrolysers supplied by renewable electricity (and in some cases through other pathways based on bioenergy) [18]. Hydrogen, as a future fuel, has a clear perspective; in the future, the price of Green hydrogen is expected to decrease from 3–4 USD/kg today to 1–1.5 USD/kg in 2050 [19]. Initially, the biggest sources of potential for hydrogen applications are the aviation [20], space [21], and shipping [22] industries. Furthermore, hydrogen, including Green hydrogen, production transportation, and storage, faces serious challenges, and the development of perfect cryogenic insulation material for LH_2_ storage is crucial, where the smallest details matter.

The purpose of this study was to evaluate the behaviour of a rigid polyurethane (PUR) foam spray that was created and applied to experimental aluminium containers that were filled with liquid nitrogen. The effect of the insulation thickness and its surface condition on cryogenic moisture uptake, effective thermal conductivity, and insulation integrity was evaluated.

## 2. Materials and Methods

### 2.1. Preparation of PUR Foam Test Specimens

#### 2.1.1. Spraying of PUR Foam

For the new qualification test, previously designed spray PUR foam [23] was chosen. Considering the technical requirements for this foam, the quantity of blowing agent Solstice LBA (Honeywell International Inc., Heverlee, Belgium) in the recipe was slightly reduced to achieve a foam density of 50 kg/m^3^.

A Reactor E-10 (Graco Inc., Maasmechelen, Belgium) high-pressure spray foam machine, equipped with a 1:1 mix ratio and Fusion™ spray gun, was used to apply the polyurethane (PUR) foam composition to experimental vessels and aluminium sheets. The used spray gun mix chamber provides an output range of about 2 kg/min for the unit. Spraying of foam was carried out at a temperature of 20 °C and air humidity of 50 ± 5% RH. At standard parameters of component heating and pressure, the cream time of the sprayed PUR composition on metal was 5 s.

To control the properties of the sprayed foam plastic, previously, 50 mm thick control panels of polyurethane foam were sprayed onto an aluminium sheet. Samples for mechanical and physical testing were cut out of these panels. PUR foam was also sprayed onto aluminium plates with dimensions of 40 mm × 40 mm × 2 mm to control the adhesion of the PUR foam to the aluminium.

#### 2.1.2. Application of PUR Foam on Experimental Vessels

For the new qualification test, experimental 19-litre aluminium vessels were chosen. Before applying foam insulation, the surface of the vessels was sandblasted with Sandblast Abrasive Grit 80. After installing bottles in a lathe-type facility, the surface of the bottles was degreased with acetone.

**Figure 1:**
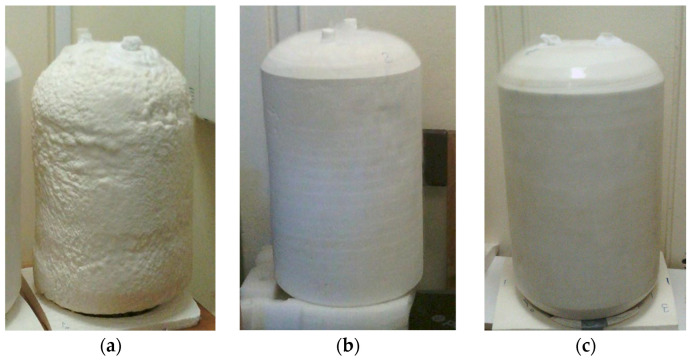
Insulated test vessels without machining (Vessel_50-R) (**a**), after machining (Vessel_40-S) (**b**), and after machining with urea coating (Vessel_55-C) (**c**).

The synchronous drive of the lathe-type facility ensured a uniform rotation speed of the vessels at 50 rpm during the application of PUR foam. Foam was spray-applied in 3–4 passes with minor interruptions, depending on the desired thickness of insulation. One of them was not mechanically processed and had a rough surface typical of sprayed foam (see Figure 1a). The other three samples were lathe-machined with a grinder to the required thickness after 24 h of foam application and had a smooth surface as depicted in Figure 1b. One of them was hand-brushed with a protective urea coating, STARFLEX T (MPM, Milano, Italy). The coating thickness was approximately 600 µm. A description of the insulated experimental vessels is listed in Table 1.

### 2.2. Characterisation of Sprayed Foam

The following test methods were used to characterise the sprayed foam. The test specimens were cut out from previously sprayed control panels.

The tensile bond strength between polyurethane foam and aluminium plates (40 mm × 40 mm × 4 mm) was used to evaluate the adhesive strength of PUR foam to aluminium in accordance with EN 1607 [24]. The thickness of the applied foam after cutting off was 20 mm. At room temperature, and after the aluminium samples with insulation applied were submerged in liquid nitrogen (LN2) and exposed for an hour, the tensile bond strength was measured. After this, the test samples were glued to aluminium plates with adapters for fixing in the test machine, like in [14]. In every mechanical testing series, eight samples were analysed.

A Static Materials Testing Machine Zwick/Roell Z010 TN (Zwick GmbH & Co., Ulm, Germany) equipped with the Basic program testXpert II was utilised for mechanical testing of PUR samples. The testing apparatus had a block for temperature adjustment and an original cryostat for testing at cryogenic temperatures (77 K), as detailed in [25].

Special equipment and foam rings with an inner diameter of 43 mm, an outer diameter of 53 mm, and a width of 13 to 14 mm were employed for the cryogenic temperature tensile test. Similar to ASTM D 2290 [26], the dependability of this foam tensile testing technique is explained in [23]. Rings were cut out in a plane perpendicular to the foam’s rising direction.

The average thermal expansion coefficient in the temperature range from 293 K to 108 K (20 to −165 °C) was determined using a vertical thermomechanical analyser TMA PT1600 (Linseis GmbH, Selb, Germany). Specimens of 4 mm × 4 mm × 20 mm were cut out perpendicularly. The cooling rate for PUR specimens was 3 K/min.

The compressive strength of PUR foams, as specified in EN 826 [27], was measured in the direction of foam rising. Cubic specimens 25 mm × 25 mm × 25 mm were cut out from the core of foam panels.

To find PUR’s thermal conductivity coefficient λ_10_, a Linseis HFM 200 thermal analyser (Linseis GmbH, Selb, Germany) was used. Along the direction of foam rise, the value of λ_10_ was measured. The samples measured 200 mm × 200 mm × 35 mm, with the top plate maintained at 20 °C and the bottom plate at 0 °C. Foam samples were kept out of direct sunlight and maintained in a room at a temperature between 20 and 22 °C while they aged.

An AccuPyc II 1340 (Micromeritics Instrument Corporation, Norcross, GA, USA) helium pycnometer was used to measure the closed-cell volume content in accordance with ISO 4590:2016 [28]. Three samples were used for each test.

#### Properties of Sprayed PUR Foam

The mechanical characteristics of spray PUR foam with a density of 50.0 ±0.5 kg/m^3^ are listed in Table 2.

Spray PUR foam with a density of 50 kg/m^3^ had good adhesion to aluminium. Its value remained practically unchanged after immersion in nitrogen. Almost all samples had a cohesive failure mode after the tensile bond test. The cryogenic resistance of the foam plastic was clearly facilitated by a sufficiently large elongation at break at cryogenic temperature (practically 5%). The compressive strength of the foam was also relatively high. The physical characteristics of spray PUR foam are listed in Table 3.

The value of the average thermal expansion coefficient in the temperature range from 293 K to 103 K is relatively large. However, a sufficiently large elongation at break at cryogenic temperature compensates for this disadvantage of PUR foam. As a result, the material withstands thermal stresses and retains the original value of adhesive strength after immersion of samples in LN_2_.

The sprayed PUR foam had a fine-cell structure similar to the previously studied PUR foam of lower density [13]. The foam’s low initial thermal conductivity coefficient value was caused by the presence of small cells, a high percentage of closed cells, and the blowing agent Solstice LBA. But as is known, during natural ageing, due to diffusion gas exchange between the foaming agent in the cells and the gases of the surrounding air, this value gradually increases. In six months, it can increase by 2–3 units.

### 2.3. Test of Insulated Vessels

After checking the properties of the PUR foam, it was applied to the experimental vessels. After mechanical processing and application of a protective coating, the vessels with foam insulation were stored in a dry room with a constant temperature of 22 °C and an air humidity of 50% RH until they reached a constant weight. After this, all the vessels were filled with LN_2_ almost to the top and weighed. Filling continued until the intensity of nitrogen evaporation, spent on cooling the aluminium vessel, sharply decreased. On average, each vessel was filled with 14 kg of LN_2_.

The vessels had two openings on top: one for filling with LN_2_, and the second for ventilation and the release of evaporating nitrogen vapours. After filling, these openings were loosely closed with foam plugs, allowing the vapours of evaporating nitrogen during the test to leak out through the gaps.

Filled, insulated vessels with leaky foam plugs on a ring-type foam base were vertically placed in an isolated, thermostatically controlled room with an air temperature of 22.0 ± 0.5 °C and a relative humidity of 80 ± 5% RH. A split-type air conditioner (Mitsubishi Electric, Budaors, Hungary) and a Residential stream humidifier (Carel S.p.A., Padova, Italy) were used to maintain a constant temperature and humidity in the air. Every hour, test vessels were weighed. During the first hour, test vessels were weighed every 10 min. For the determination of cryogenic moisture uptake, insulated test vessels were weighed immediately after all the nitrogen had evaporated.

For the control of PUR foam insulation integrity and quality, a thermal imaging FLIR IR Camera E 54 (FLIR Systems, Inc., Tallinn, Estonia) was used. Additionally, the insulation surface temperature of the filled vessels was controlled using a special surface thermocouple and a digital thermometer GMH 3210 (Greisinger electronic GmbH, Regenstauf, Germany).

Every test specimen underwent a maximum of three repeat runs. The specimens were reconditioned in a dry air-filled chamber for a few days in between runs until the weight stabilised. Almost all of the water absorbed during the experiment was evaporated as the vessels’ weight was the same as at the beginning of the experiment. After that, the vessels were filled with LN_2_.

## 3. Results and Discussion

**Figure 2:**
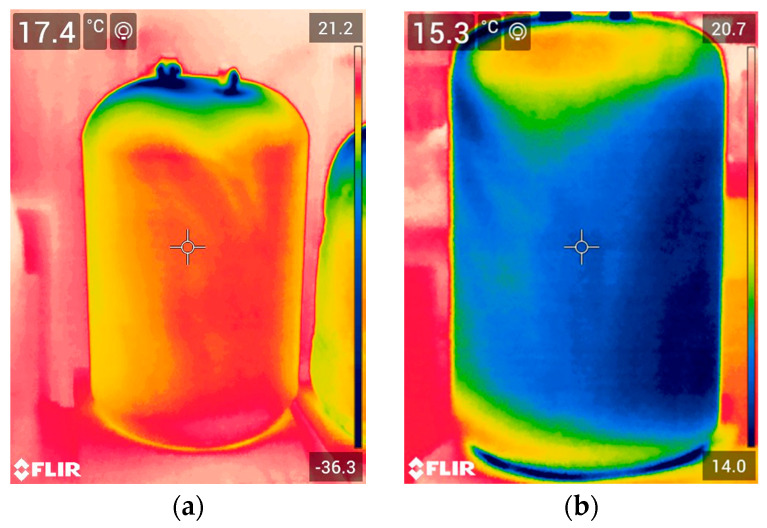
FLIR IR thermal images of full (**a**) and partially emptied (**b**) insulated vessels with LN_2_.

Filling an insulated vessel with liquefied gas is a quality test for any cryogenic insulation. All insulated vessels passed this test and did so repeatedly. During the filling of insulated test vessels, no crackling sound was heard. Both visually and using IR thermal images, control of filled insulated vessels also reveals no defects in the insulated test vessels. Typical IR thermal images of tested vessels filled with LN_2_ are presented in Figure 2. The rest of IR images are in Figures S1–S5.

As can be seen at the beginning of the test, when the vessels were completely filled, the lower temperature of the upper part of the insulated vessel’s surface allowed air moisture to condense through LN_2_ evaporation through two non-hermetic, plugged holes. When a significant portion of the LN_2_ in the vessel evaporated, the temperature of the upper part of the PUR foam insulation surface became slightly higher than the temperature of the insulation surface of the lower part of the vessel, still filled with LN_2_. This was due to the fact that the upper part of the inner surface was no longer in contact with liquid nitrogen, but only with its vapours.

The surface temperature of the foam insulation of the tanks filled with LN_2_ gradually dropped several degrees lower over the course of an hour, compared to the ambient air temperature (22.0 °C). The readings of thermometers with different operating principles are given in Table 4. There are average values of the temperature at three fillings. Temperatures were measured 1 h after filling of bottles, when, in our opinion, a stable temperature regime was established in an insulated, thermostatically controlled room.

The temperatures measured by the thermocouple differ from those measured by the FLIR IR Camera, because the correct value of the emissivity coefficient for foam surfaces for PUR has not yet been established for the FLIR IR Camera. However, this does not change the trend. The lowest surface temperature was observed in Vessel_40-S, which had the thinnest insulation thickness. The highest surface temperature was found in Vessel_60-S, which had the greatest insulation thickness.

The surface temperature of all insulated vessels was lower than the dew point (18.9 °C) of air with 80% RH. Therefore, air moisture was condensed on the insulation surface. Before weighing the bottles, drops of condensed water were removed with filter paper.

The average amount of moisture absorbed by the PUR foam insulation of the vessels during evaporation of LN_2_ from three tests is given in Table 5. The higher quantity of absorbed moisture by Vessel_60-S may be explained by the longest time of liquid nitrogen evaporation from the test vessels (34 h). Vessel_50-R also had a higher value of moisture uptake because it was difficult to remove condensed drops of water from the uneven surface of the foam.

However, considering the thickness and mass of the applied PUR foam, the insulation of vessel B, which had the smallest thickness, exhibited the greatest cryogenic moisture uptake (10.4%). The insulation of Vessel_60-S, with the greatest thickness, adsorbed less moisture in percentage terms than other vessels without protective coatings. This is in a certain agreement with the data of [29], which indicates that the moisture adsorbed by the insulation is primarily concentrated in the layer closest to the surface of the foam insulation. Only a small amount of moisture penetrates deep into the insulation layer.

The insulation of test Vessel_55-C, covered with urea coating, practically did not absorb moisture. On the other hand, on the smooth surface of the coating, the moisture in the air condensed well, and drops of it flowed down the vertical surface during the test.

Since all foam insulation materials are somewhat permeable to gases and water vapours, even high-quality, closed-cell polyurethane foams can retain water in actual insulated vessel applications. The accumulation of water due to water vapour driving into the foam in the presence of a temperature gradient, as well as the thermophysical transport mechanisms of vapour drive, condensation, and ice formation within foam insulation subjected to cold boundary conditions, are covered in [18,19] and other sources. In our instance, the absorption of water condensed on the PUR foam insulation’s surface complemented the effects of the gas permeability of the foam and the condensation of water vapour inside the foam.

Since the duration of the experiment was relatively short and the accumulated amount of moisture was not so great, after the test, all absorbed moisture evaporated within three days at room temperature and an air humidity of 50% RH. When the insulated vessel was refilled with LN_2_, the insulated vessel had the same mass as before the first test. With this duration of the experiment, no cumulative effect of moisture accumulation was observed.

When filling the test vessel with LN_2_, the aluminium vessel first cooled rapidly, accompanied by violent boiling of the LN_2_. With an average mass of these containers of 3.57 kg and an average heat capacity of aluminium of 800 J/(kg∙K) in the temperature range of 77–293 K, at least 3.2 kg of LN_2_ was required to cool the vessel. Filling the vessel, accompanied by boiling of nitrogen, continued until the intensity of nitrogen evaporation, spent on cooling and filling the aluminium vessel, sharply decreased. On average, each vessel was filled with 14 kg of LN_2_. After placing the filled vessel in a special room, periodic weighing of the vessels with continuously evaporating LN_2_ began.

**Figure 3:**
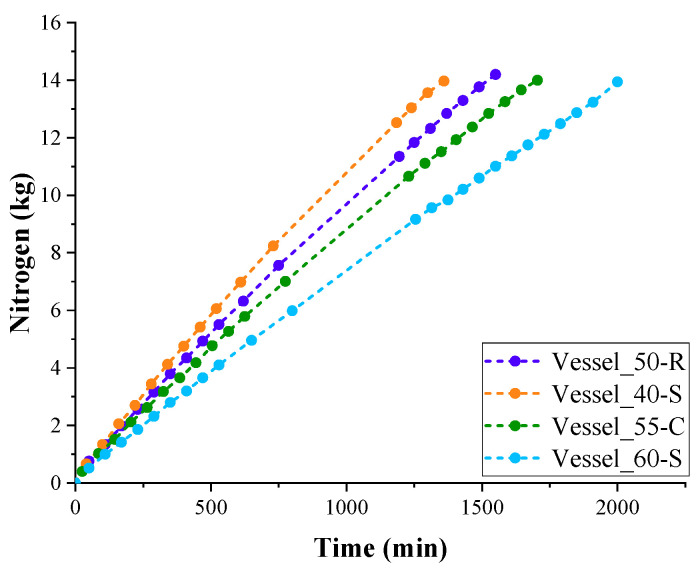
LN_2_ evaporation curves of insulated test vessels.

LN_2_ evaporation curves of insulated test vessels are presented in Figure 3. The evaporation rate of LN_2_ in test Vessel_40-S with thinner PUR foam insulation was highest. The lowest evaporation rate of LN_2_ was observed in test Vessel_60-S with the highest PUR foam insulation thickness.

The filling experiments with PUR foam vessels were repeated three times. The average values of evaporation time of LN_2_ from the tested vessels filled with 14 kg of LN_2_ are listed in Table 6. As can be seen, the evaporation times of LN_2_ from Vessel_50-R and Vessel_55-C with insulation thicknesses of 50 and 55 mm occupy an intermediate position between the evaporation times of LN_2_ from Vessel_40-S and Vessel_60-S.

**Figure 4:**
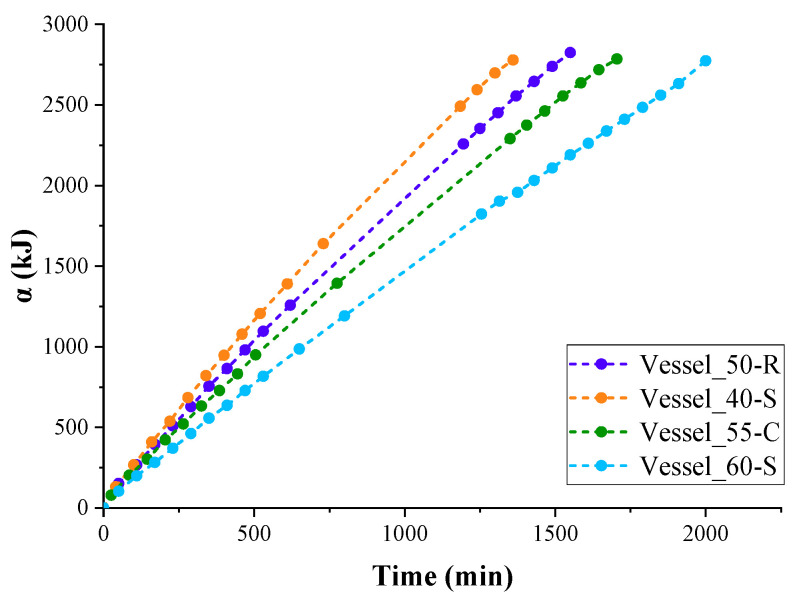
LN_2_ heat absorption curves of insulated test vessels.

LN_2_ receives all the heat necessary for evaporation from the surrounding air due to thermal conductivity, convection, and radiation of air. The total amount of heat received by LN_2_ from the surrounding air and transferred by the PUR foam thermal insulation can be calculated when the heat of nitrogen vaporisation is known (5.57 kJ/mol or 198.9 kJ/kg). The curves of the total absorbed heat depending on the time of LN_2_ boil-off are shown in Figure 4.

As follows from the data shown in Figure 3, the rate of evaporation of LN_2_ gradually decreases as the vessels are emptied. Accordingly, the rate of absorption of heat spent on the evaporation of nitrogen decreased (Figure 4). This occurred because the value of the heat flow absorbed by LN_2_ during boiling, in relation to the evaporation surface, decreased as the liquid level in the container decreased.

Additionally, at the beginning of the experiment, all the heat transferred through the insulation and the vessel wall was transferred to the liquid, which had a high thermal conductivity coefficient. Over time, as the vessels emptied, an increasing portion of the heat was transferred to gaseous nitrogen vapours, which have a much lower thermal conductivity. Accordingly, the nitrogen vapours did not heat up as quickly as the liquid due to convection, and further evaporation of nitrogen in the container with saturated nitrogen vapours slowed down.

Analysis of both LN_2_ evaporation curves and absorption curves showed that second-degree polynomials approximate all experimental curves with a reliability of at least 0.98. Taking this into account, it was possible to calculate the value of the thermal conductivity coefficient of insulation by interpolating the value of the heat flow rate for the first minutes of the experiment, when almost the entire surfaces of both vessels and the insulation participated in the heat exchange process.

In shape, our vessel consisted of a cylinder and two hemispheres. Accordingly, it can be assumed that in the heat exchange process, the effective heat transfer area consists of the surface of the cylinder and the sphere. According to Fourier’s law, the heat flow in the insulation layer is directed from the hot surface to the cold one and is calculated using the thermal conductivity coefficient of the material, its geometric parameters, and the temperature difference. For a cylindrical surface, the heat flow rate (Q) is calculated using formula (1) [30]:

(1) $$Q=\frac{2\pi \lambda L(T_{1}-T_{2})}{ln\frac{d_{2}}{d_{1}}}$$

Equation (1) shows that the temperature changes logarithmically across the thickness of a cylindrical wall, unlike a flat wall, where its profile is linear. This is because, with an increasing radius, the surface through which heat is transferred increases; i.e., the specific heat flow (related to the area) decreases with increasing radius.

The temperature field in a spherical wall with a constant thermal conductivity coefficient obeys the hyperbolic law. Therefore, the heat flow rate passing through a spherical insulation, W, is equal to (2) [31]:

(2) $$Q=\frac{2\pi \lambda (T_{1}-T_{2})}{\frac{1}{d_{1}}-\frac{1}{d_{2}}}$$

Summing these two flows, we obtain Equation (3):

(3) $$Q=2\pi \lambda (T_{1}-T_{2})(\frac{L}{ln\frac{d_{2}}{d_{1}}}+\frac{1}{\frac{1}{d_{1}}-\frac{1}{d_{2}}})$$

where *Q*—heat flow rate, *λ*—thermal conductivity coefficient, *d*_1_—internal diameter of cylinder and sphere, *d*_2_—outer diameter of cylinder and sphere, *L*—length of cylinder, and *T*_1_ and *T*_2_—temperature of internal and outer surfaces of insulation.

**Figure 5:**
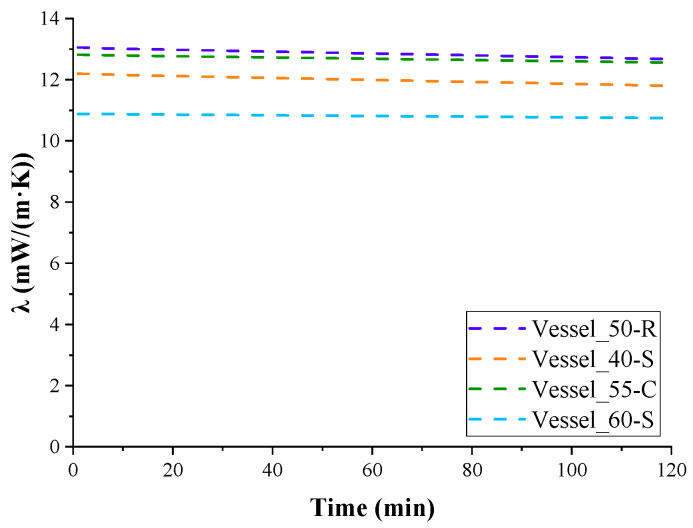
Changes in effective thermal conductivity coefficient of tested vessel’s insulation with LN_2_ evaporation time.

The thermal conductivity coefficient of the insulation is easily calculated from this equation. Figure 5 shows the change in the average calculated values of the thermal conductivity coefficient for three experiments, excluding the variation in contact area with LN_2_. However, when interpolating the heat absorption curve data (Figure 4) to the first minute of the experiment, when LN_2_ contacts almost the entire surface of the vessel, the obtained thermal conductivity coefficient can be considered the effective thermal conductivity coefficient of the insulation in the temperature range 77–293 K.

The average values of the thermal conductivity coefficients for the insulation of the tested vessels, based on three measurements, are given in Table 7. Since the total duration of all experiments did not exceed 3 weeks, no tendency for the thermal conductivity coefficients of the insulation to increase due to ageing of foam was observed. No cumulative effect of cryogenic moisture uptake was observed either. Therefore, the deviations of the measured and calculated indicators were within the experimental error.

As can be seen, the lowest values of the thermal conductivity coefficient were observed in the insulation of test Vessel_60-S, which had the greatest thickness of insulation and, consequently, the smallest cryogenic moisture uptake in percentage of the total mass of insulation among insulated vessels without a protective coating.

The highest value of the effective coefficient of thermal conductivity was obtained for the insulation of Vessel_50-R, which had a denser, rough surface integral layer and, accordingly, a greater integral density of PUR foam insulation compared to the others, in which this layer was removed during processing.

**Figure 6:**
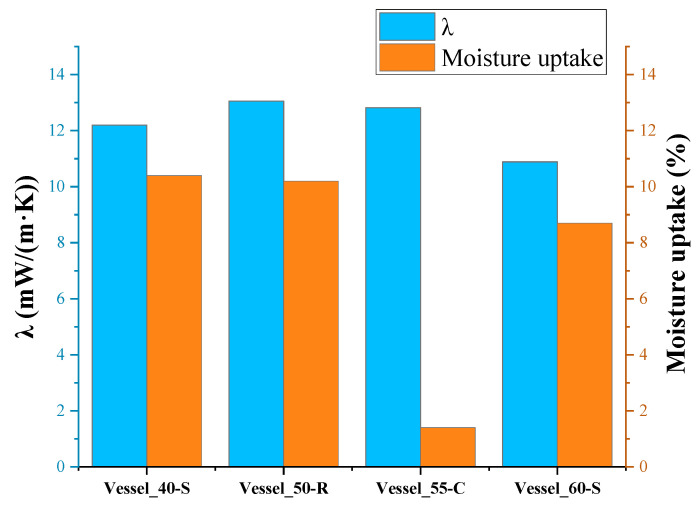
Effective thermal conductivity coefficient and cryogenic moisture uptake of insulated vessels.

The effective thermal conductivity coefficient of the insulation of Vessel_50-R was higher than the effective thermal conductivity coefficient of the insulation of Vessel_60-S, apparently due to the greater effect of the cryogenic moisture uptake (Figure 6), which amounted to 10.4% of the total mass of the insulation. The insulation of Vessel_55-C had an even greater coefficient. Despite this, thanks to the protective coating, the insulation almost did not absorb moisture. However, the moisture condensed very intensely on the surface of the coating due to the greater heat capacity of the coating, which was cooled by several degrees. Drops of moisture flowed down from the smooth vertical coating, but its contribution to thermal conductivity was obviously significant. Additionally, due to the cooling of the coating, heat from the environment entered the insulation not only through air convection but also through the thermal conductivity of the coating material.

## 4. Conclusions

A boil-off test was conducted for experimental aluminium vessels with spray-applied PUR foam insulation of varying thicknesses and surface conditions. During all operations of filling the vessels with LN_2_ and conducting the experiment, the integrity of the PUR foam remained. No defects or cracks in the insulation were revealed.

By interpolating the data of the dynamic boil-off experiment, it is possible to determine the value of the effective thermal conductivity coefficient of the insulation in the corresponding temperature range. The values of this coefficient depend on the thickness of the insulation and cryogenic moisture uptake, which is directly dependent on the temperature and humidity of the surrounding air. The lowest thermal conductivity coefficient was found in the vessel with the thickest insulation (Vessel_60-S), which had the lowest moisture uptake (percentage) among insulations without a protective coating. Insulation of the vessel with a protective coating (Vessel_55-C) had a minimum moisture uptake; however, due to the thermal conductivity of the coating material and the intense condensation of moisture on its surface, the value of the thermal conductivity coefficient was not minimal.

To explore the cryogenic moisture uptake processes in more depth, it is necessary to study this phenomenon during longer exposures in future. It is necessary to conduct other experiments with a constant replenishment of the experimental vessels with LN_2_, without pauses, to continuously maintain the temperature gradient between inner and outer side of insulation.

## Figures and Tables

**Table 1:** Insulated experimental vessels.

Vessels	Foam Thickness, mm	Foam Surface
Vessel_40-S	40	Smooth (after machining)
Vessel_50-R	50	Rough (without machining)
Vessel_55-C	55	Smooth and urea coating
Vessel_60-S	60	Smooth (after machining)

**Table 2:** Mechanical properties of spray PUR foam.

Property	Unit	Average	Standard Deviation
Initial tensile bond strength	MPa	0.44	0.07
Tensile bond strength after immersion in LN_2_	MPa	0.46	0.06
Tensile strength at 77 K	MPa	0.88	0.09
Elongation at break at 77 K	%	4.97	0.23
Compressive strength	MPa	0.20	0.01

**Table 3:** Physical properties of spray PUR foam.

Property	Unit	Average	Standard Deviation
Average thermal expansion coefficient (293–108 K)	K^−1^·10^6^	0.84	0.05
Volume content of closed cells	%	96.5	0.2
Thermal conductivity coefficient λ_10_ after 1 day	mW/(m·K)	16.1	0.2
Thermal conductivity coefficient λ_10_ after 7 days	mW/(m·K)	16.6	0.2

**Table 4:** The surface temperature of filled vessels (°C).

	Test Vessels			
Thermometer	Vessel_40-S	Vessel_50-R	Vessel_55-C	Vessel_60-S
Thermocouple	16.5 ± 0.2	16.9 ± 0.3	16.8 ± 0.2	17.2 ± 0.4
FLIR	14.2 ± 0.4	14.9 ± 0.6	15.6 ± 0.6	15.9 ± 0.5

**Table 5:** Cryogenic moisture uptake of insulated test vessels.

	Test Vessels			
Test	Vessel_40-S	Vessel_50-R	Vessel_55-C	Vessel_60-S
Moisture uptake, g	149	173	33	173
Moisture uptake, %	10.4	10.2	1.4	8.7

**Table 6:** Evaporation time of LN2 from tested insulated vessels.

Vessels	Foam Thickness, mm	Evaporation Time, h
Vessel_40-S	40	23.5
Vessel_50-R	50	26.2
Vessel_55-C	55	28.3
Vessel_60-S	60	33.8

**Table 7:** Effective thermal conductivity coefficient of vessels’ insulation.

	Test Vessels			
Thermal Conductivity	Vessel_40-S	Vessel_50-R	Vessel_55-C	Vessel_60-S
λ_77–293_, mW/(m·K)	12.2 ± 0.2	13.1 ± 0.1	12.8 ± 0.1	10.9 ± 0.2

## Data Availability

The authors state that the presented data will be made available on request by email.

## Supplementary Materials

The following supporting information can be downloaded at: https://www.mdpi.com/article/10.3390/polym17121669/s1, Figure S1: IR thermal image (a) and visual image (b) of insulated Vessel_50-R.; Figure S2: IR thermal image (a) and visual image (b) of insulated Vessel_40-S.; Figure S3: IR thermal image (a) and visual image (b) of insulated Vessel_55-C.; Figure S4: IR thermal image (a) and visual image (b) of insulated Vessel_60-S; Figure S5: Vessel preparation process: (a) test vessel before spraying; (b–c) spraying process; (d) ready insulated test vessel (Vessel_50-R); (e) test vessel after machining (Vessel_40-S); (f) test vessel with urea coating (Vessel_55-C).

## Abbreviations

The following abbreviations are used in this manuscript:

PUR	Polyurethane
RH	Relative humidity
LO_2_	Liquid oxygen
LH_2_	Liquid hydrogen
LN_2_	Liquid nitrogen
λ_10_	Thermal conductivity coefficient
FLIR	Forward-looking infrared radiation
