# Supplementary material for: Influence of Rigid Polyurethane Foam Production Technology on Cryogenic Water Uptake
Source: Polymers (Basel). 2025 Jun 16;17(12):1669. doi: 10.3390/polym17121669 (PMC12196953; doi:10.3390/polym17121669)
Supplement: Supplementary file 1 [file polymers-17-01669-s001.zip › polymers-3702275-supplementary.pdf]

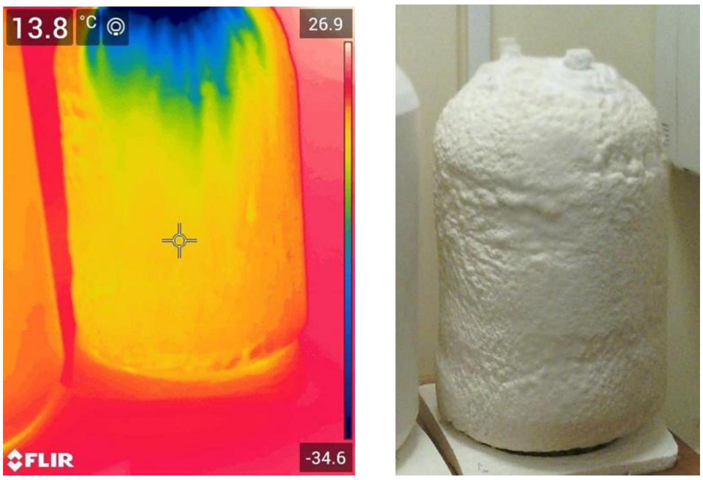

(**a**) (**b**)

Figure S1. IR thermal image (**a**) and visual image (**b**) of insulated Vessel_50-R.

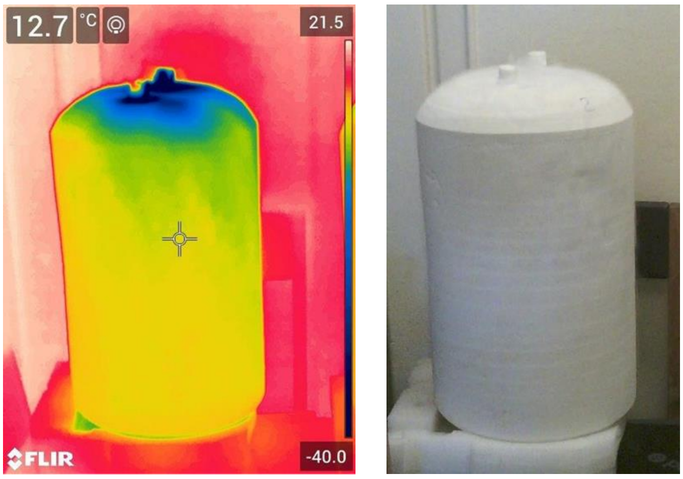

(**a**) (**b**)

Figure S2. IR thermal image (**a**) and visual image (**b**) of insulated Vessel_40-S.

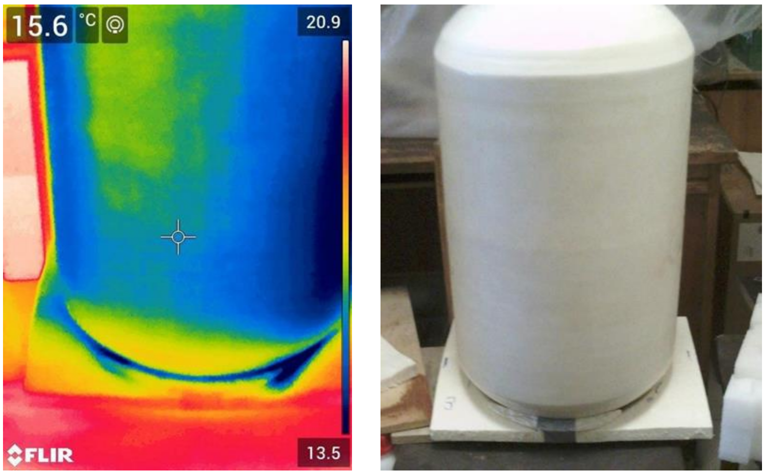

(**a**) (**b**)

Figure S3. IR thermal image (**a**) and visual image (**b**) of insulated Vessel_55-C.

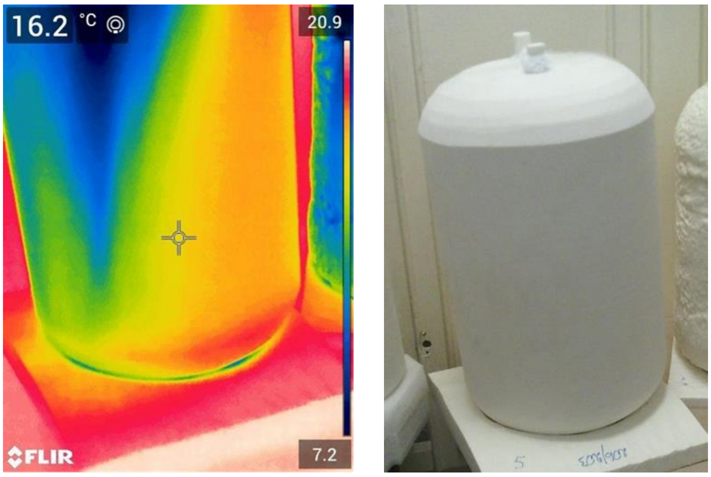

(**a**) (**b**)

Figure S4. IR thermal image (**a**) and visual image (**b**) of insulated Vessel_60-S.

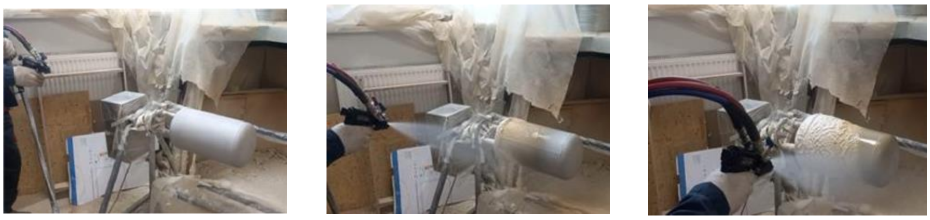

(**a**) (**b**) (**c**)

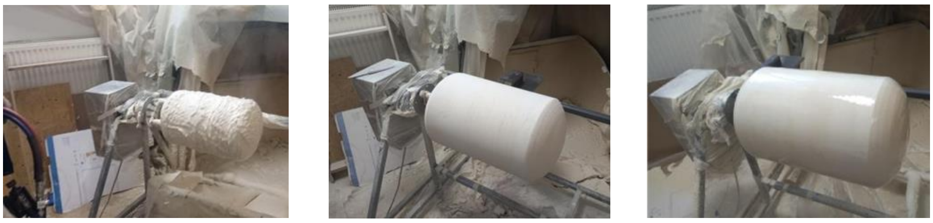

(**d**) (**e**) (**f**)

Figure S5. Vessel preparation process: (**a**) Test vessel before spraying; (**b-c**) Spraying process; (**d**) Ready insulated test vessel (Vessel_50-R); (**e**) Test vessel after machining (Vessel_40-S); (**f**) Test vessel with urea coating (Vessel_55-C).
